# Electro-Freezing of Supercooled Water Is Induced by Hydrated Al^3+^ and Mg^2+^ Ions: Experimental and Theoretical Studies

**DOI:** 10.1021/jacs.3c05004

**Published:** 2023-08-21

**Authors:** Leah Fuhrman Javitt, Surajit Kalita, Kshatresh Dutta Dubey, David Ehre, Sason Shaik, Meir Lahav, Igor Lubomirsky

**Affiliations:** † Department of Molecular Chemistry and Materials Science, Weizmann Institute of Science, Rehovot 7610001, Israel; ‡ Institute of Chemistry, The Hebrew University of Jerusalem, Edmond J. Safra Campus, Givat Ram, Jerusalem 9190401, Israel; § Department of Chemistry, School of Natural Sciences, Shiv Nadar Institution of Eminence, Greater Noida, Uttar Pradesh 201314, India

## Abstract

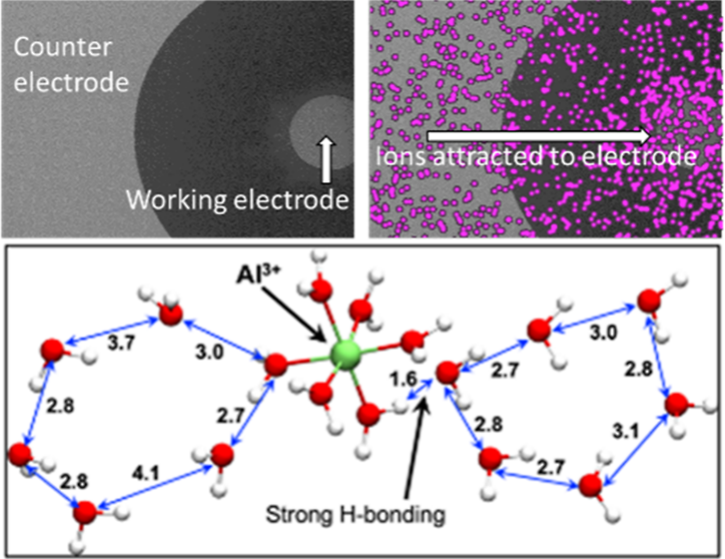

This work reports that the octahedral hydrated Al^3+^ and Mg^2+^ ions operate within electrolytic cells as kosmotropic (long-range order-making) “ice makers” of supercooled water (SCW). 10^–5^ M solutions of hydrated Al^3+^ and Mg^2+^ ions each trigger, near the cathode (−20 ± 5 V), electro-freezing of SCW at −4 °C. The hydrated Al^3+^ ions do so with 100% efficiency, whereas the Mg^2+^ ions induce icing with 40% efficiency. In contrast, hydrated Na^+^ ions, under the same experimental conditions, do not induce icing differently than pure water. As such, our study shows that the role played by Al^3+^ and Mg^2+^ ions in water electro-freezing is impacted by two synchronous effects: (1) a geometric effect due to the octahedral packing of the coordinated water molecules around the metallic ions, and (2) the degree of polarization which these two ions induce and thereby acidify the coordinated water molecules, which in turn imparts them with an ice-like structure. Long-duration molecular dynamics (MD) simulations of the Al^3+^ and Mg^2+^ indeed reveal the formation of “ice-like” hexagons in the vicinity of these ions. Furthermore, the MD shows that these hexagons and the electric fields of the coordinate water molecules give rise to ultimate icing. As such, the MD simulations provide a rational explanation for the order-making properties of these ions during electro-freezing.

## Introduction

Water molecules in pure bulk water at pH 7 can self-assemble into numerous hydrogen bonding architectures. However, in order for ice nucleation to occur, the water molecules must be arranged as “ice-like” hexagons. In bulk water, the hexagons represent a minority with respect to the many other architectures.^1^ This minimizes the probability of getting an ice nucleus above the critical size for ice nucleation. Consequently, water can be supercooled homogeneously down to −48 °C without freezing.^2^

The temperature of icing of the supercooled water (SCW) is vital in many processes. Climate control, rain precipitation, and food preservation are a few of these processes.^3^ This icing temperature may be controlled heterogeneously by performing the icing experiments on surfaces or by the application of electric fields (electro-freezing).^4^

Recently, we demonstrated on the charged surfaces of pyroelectric crystals that the icing temperature of SCW can be influenced by an electrochemical process.^5^ In the presence of a pyroelectric field, upon cooling, hydrogen-bonding trigonal-planar ions are attracted and concentrated at the charged hemihedral faces. There, they stabilize hexagonal “ice-like nuclei”. These ions replace some of the dissociable hydrogen bonds of hexagonal-ordered water with covalent bonds. This stability leads to an elevation in the freezing temperature of SCW.^5c^

Based on those findings, we hypothesized that electro-freezing can also be influenced by ions with other hydration geometries that have the ability to form and stabilize hydrogen bonds with bulk water molecules. In particular, we considered the metallic ions, which are known as Lewis acids, that interact with the lone pair electrons of the water molecules to form stable hydrates.^6^ Those hydrates might assume various structures: tetrahedral, octahedral, square antiprism, or tricapped trigonal prism.^7^ The structure of the water molecules surrounding the hydrated metal cation will be determined by the structure of the hydrated ion. Additionally, the coordinated water molecules undergo polarization by the metal. The polarization depends on the square of the valency of the ions and the volume of the ion’s nuclei.^8^ Consequently, the Me···O- bond length and strength between the metal and the coordinated water are determined by the degree of polarizability.^9^ In hydrated metal complexes, the coordinated water molecules are acidic, and according to the Bronsted–Lowry principle, the bordering interfacial water molecules can operate as conjugated bases.^6^ Subsequently, the acid–base interactions between the water molecules in the vicinity of the hydrated ions are stronger than the interactions between water molecules at pH 7 in pure water. Therefore, if the hydrated ions induce “ice-like” aggregates in their vicinity, they should be further stabilized by strong acid–base interactions and raise the icing temperature of SCW. Furthermore, if the icing occurs next to a cathode, where hydroxide ions are formed as a result of the electrolysis of water, there may be an even stronger stabilization due to the acid–base interactions.

Here, we demonstrate by experiment and molecular dynamics (MD) simulations that the octahedral hydrated ions of Al^3+^ and Mg^2+^ induce the formation of “ice-like” hexagons and thus operate as “ice makers” when concentrated near the cathodes of electrolytic cells.

## Experimental Results

In order to investigate the hypothesis that the polarizability of an ion plays a role in its ability to act as an “ice-maker” during electro-freezing, and to disentangle this effect from the geometric effect, we selected the third-row metals of the periodic table. The ions Al^3+^, Mg^2+^, and Na^+^ can be expected to have similar geometry in the presence of bulk water; however, they differ in valency and ionic radius, which translates to an increase in polarizing power along the row (see Table 1).

**Table 1:** Comparison between Al^3+^, Mg^2+^, and Na^+^ ions: coordination, M–O Bond Length, Ionic Radius, Polarizing Power , First Shell Lifetime, and the Number of Water Molecules in the Second Shell

ion	coordination	M–O bond length [Å]	ionic radius [Å]	polarizing power	first shell lifetime [s]	number of water molecules in the 2nd shell
Al^3+^	octahedral^7a^	1.89^10^	0.53^11^	10	6.3^12^	12^12b^
Mg^2+^	octahedral^7a^	2.1^10^	0.72^11^	3.9	10^–6^ ^10,12b^	12^12b^
Na^1+^	octahedral^7a^	2.43^13^	0.1^11^	1	10^–12^ ^10^	12.4^12b^

As a result of this difference, diffraction, scattering, and spectroscopic methods, as well as theoretical calculations, also show differences in the strength of the binding of the coordinated water molecules with neighboring water molecules.^7,10−14^ Furthermore, due to the high polarizing power of the Al^3+^ ion, one can observe the formation of a second stable water shell.^10,15^ A less rigid water layer was reported near the Mg^2+^ ion.^10,14b,16^ On the other hand, the monovalent Na^+^ hydrated ions weaken the hydrogen bond interactions with neighboring water molecules in comparison to those in bulk water.^10^

Solutions of the nitrate complexes of these ions at various concentrations were made, and their icing properties were investigated on pyroelectric surfaces (LiTaO_3_). No differences in icing temperatures were found compared to pure water. However, the field on pyroelectric crystals is at least 2 orders of magnitude lower than what can be applied in electrolytic cells.^5d,17^

**Figure 1:**
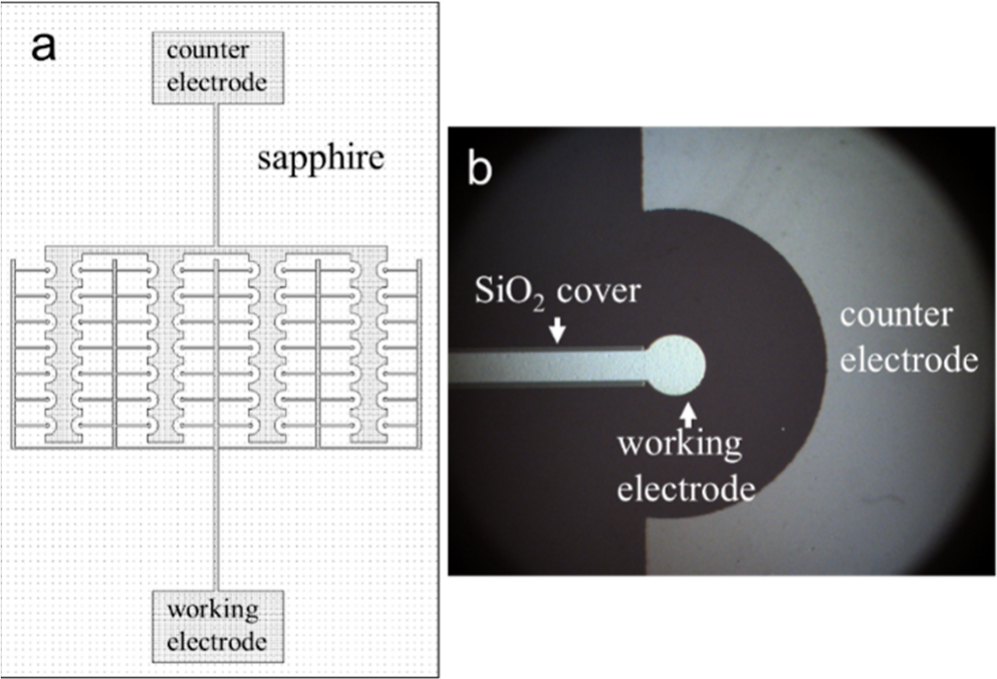
(a) Design of the planar asymmetric electrode design. (b) Optical microscopy image of one pair of anode and cathode with an insulating layer. Both the working and counter electrodes are made of nickel. The working electrode has a 50 μm radius, with 200 μm between the working and counter electrodes. A 1 μL droplet was placed, covering four anode–cathode pairs.

**Figure 2:**
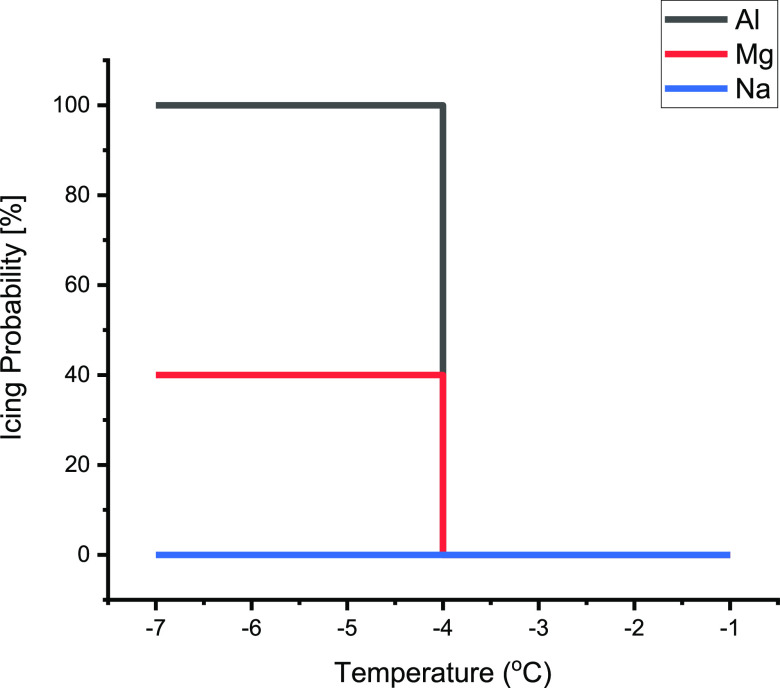
Icing temperature of Al^3+^, Mg^2+^, Na ^+^, and pure water. Al^3+^ freezes SCW at −4 °C with 100% probability, Mg^2+^ freezes SCW at −4 °C with 40% probability, Na^+^ does not affect electro-freezing, and pure water freezes with voltage at −12 °C.

To solve this problem, icing experiments with these ions were performed on metallic electrodes where much higher fields could be applied. The electrode is required to be a metal with low solubility in water which does not freeze SCW at an elevated temperature and has a native oxide with relatively high electric conductivity. After screening several metals, Ni electrodes were selected as they were not observed to affect the icing process of pure water at the voltages used for the experiments. Experiments were performed on a series of large and small electrodes, as seen in Figure 1. Solutions were cooled using a Peltier cooling stage. The temperature is accurate at 0.5 °C. First, linear sweep voltammetry at −5 °C was performed on a solution of 10^–5^ M Al(NO_3_)_3_ and icing was observed at −20 ± 5 V. In order to investigate the icing temperature, a pulse of −50 V was applied to the small electrode, which concentrated the charge and attracted the positively charged metal cation. This voltage was seen as high enough and well above the range that would cause variations in the experiments. In the absence of an electric field, 10^–5^ M solutions of Al(NO_3_)_3_ freeze at −21.5 ± 1.5 °C, 10^–5^ M solutions of Mg(NO_3_)_2_ freeze at −19.5 ± 1.5 °C, and 10^–5^ M solutions of Na(NO_3_) freeze at −21 ± 1 °C. Pure water freezes at −18 °C ± 2 °C. However, in the presence of an electric field, as seen in Figure 2, pure water freezes consistently on Ni electrodes at a temperature of −12 °C, while 10^–5^ M Al^3+^ solutions freeze below −4 °C 100% (30 of 30 experiments). These results show that Al^3+^ has the ability to act as an efficient kosmotropic (long-range order-inducing) “ice-maker”.

In order to investigate the effect of polarization on “ice-making” ability, we selected hydrated ions with the same octahedral configuration of the coordinated water molecules, but with different sizes and valencies. Mg^2+^ ions are similar to Al^3+^ ions; however, the M–O bond length is 2.1 Å^10^ (versus 1.89 Å^10^), which is still much shorter than the 2.89 Å O–O bond in bulk water.^10^ The lifetime of the water molecules surrounding the Mg^2+^ cations is 10^–6^ s^12b^ (vs 6.3 s for Al^3+^^12^), which is 6 orders of magnitude greater than the lifetime of the water surrounding the Na^+^ cation.^10^ We found that 10^–5^ M Mg^2+^ solutions freeze below −4 °C 40% (18/45), and this probability remains constant down to −8 °C, where pure water can begin to undergo electro-freezing (see the Supporting Information). 10^–5^ M Na^+^ solutions do not induce icing at any temperature (0/25).

These results suggest that the ability of a cation to polarize the surrounding water molecules is an important factor in its ability to induce icing. The fact that the freezing onsets sharply at −4 °C, for Mg and for Al but with different probabilities implies that the same species are responsible for nucleation, but for Al, they are formed always and for Mg with a probability of 40% only.

In order to provide the support that ion-induced “ice-like” hexagons are responsible for the ice nucleation, independent MD simulations were performed.

## MD Simulations

According to prior computational studies, the strength of the electric field necessary for the ice nucleation process can reach up to 1.0 V/Å.^4a,4d,4f,18^ This value, however, is higher than the experimentally reported one.^4b,4c,4e,17,19^ As a result, the electric field we optimized in our study is of a moderate magnitude. We began our investigation with a variable electric field that ranged from 0.1 to 0.5 V/Å. These field values were applied to three trial systems, which differed in their water densities within the MD simulation box. Preliminary results from all the performed simulations indicated that ice nucleation on the surface of Al^3+^ ions in the presence of an electric field requires a specific density of water and a minimum strength of the external electric field. Thus, we found that the ice nucleation process occurred best in a system with 710 water molecules (density 0.99 g/cm^3^) and an external electric field of strength 0.2 V/Å as well as 0.5 V/Å, depending on the simulation temperature. The melting point of real water is 273.15 K, and that of the TIP4P/2005 water model is recorded at ∼250 K. Thus, we carried out our simulations at both temperatures and interestingly observed a similar event except for the alteration of the external electric field’s strength. We note that the rise in temperature by 23° (250 → 273 K) requires us to increase the strength of the external electric field by 150% (0.2 → 0.5 V/Å) to observe the icing phenomenon. It further indicates that the external electric field plays a very crucial role in organizing the water molecules into ice-like structures. Nevertheless, it needs to be mentioned that the strength of the applied external electric field is much smaller than the generated local electric field (LEF) by the ice structures (0.2 V/Å [applied] vs 1.5 and 1.0 V/Å [LEF]). Table 2 shows a detailed description of LEF calculations.

**Table 2:** LEF Generated at the Centers of the Hexagons (see Figures 5 and 6) by the [Al(H_2_O)_6_]^3+^(H_2_O)_6_(H_2_O)_5_ and [Mg(H_2_O)_6_]^2+^(H_2_O)_6_(H_2_O) Unit at the Starred Points of the Nucleating Ice Crystals^a^

		electric field (V/Å)			
positions/points		*x*-component	*y*-component	*z*-component	total
for Al^3+^	1	–1.373	–0.426	–0.404	1.493
	2	0.912	–0.511	–0.281	1.083
for Mg^2+^		–1.076	–0.0365	0.181	1.0918

a During quantification of LEF, we consider an Al^3+^/Mg^2+^···H_2_O bond as a vector quantity (note that the considered bond vector is closely parallel to the *x*-axis). LEF calculations are performed by the TITAN program.^21^

In order to explore the ice-making power of Mg^2+^ ions, similarly, we first determined the system that was found to have the appropriate density for ice formation (cf. the Computational Methodology section for detailed discussion).

### Nucleation Centers of Ice

**Figure 3:**
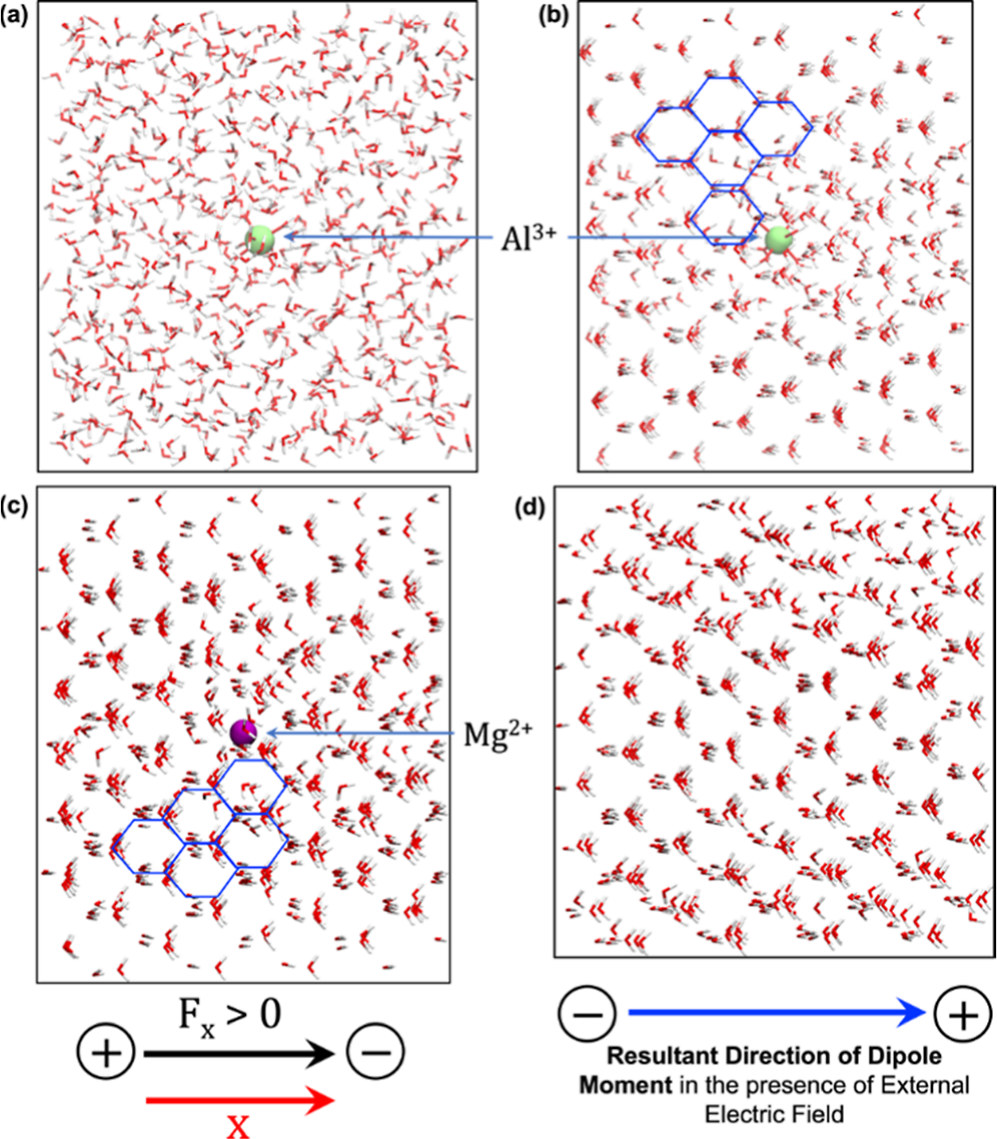
Depiction of the water simulation box: (a) initial conformation under an electric field, (b) icing conformation obtained after 10 ns of simulation time in the presence of Al^3+^ ions, (c) icing conformation obtained after 14 ns of simulation time in the presence of Mg^2+^ ions, (d) conformation obtained after 150 ns of simulation time in the absence of Al^3+^ and Mg^2+^ ions. The external electric field is applied along the *x*-axis. The red arrow represents the cartesian axis, and the black arrow denotes the direction of a positively applied external electric field. The blue arrow shows the direction of the dipole moment in the presence of an external electric field. Blue hexagons are drawn for better visualization of the ice hexagons.

After optimizing the strength of the external electric field (0.2 V/Å at 250 K) and ascertaining the water density that promotes nucleation, we divided our investigation into two parts (Figure 3a–d): in the presence of metal ions (Al^3+^ and Mg^2+^) and in the absence of those ions. Our findings show that in the presence of the Al^3+^ion (Figure 3a), water molecules started to arrange themselves in the hexagonal configuration after just 5 ns and achieved the geometry depicted in Figure 3b within only 10 ns. Similarly, in the presence of the Mg^2+^ ion, the corresponding water molecules organized themselves within 8–9 ns and completed the icing process at 14–15 ns (cf. Figure 3c). Further extension of our simulation to 500 ns showed no conformational changes for both systems (see the RMSD plot in Figure S2a,b in the Supporting Information). This observation further reveals that the time scale of icing in the presence of Mg^2+^ is somewhat longer by ∼4 to 5 ns than that for Al^3+^, which reflects the slower rate of ice formation in the presence of the Mg^2+^ ion than that of the Al^3+^ one. It should be noted that ice structures become stable once we remove the electric field after electro-freezing occurs. A detailed description along with the statistical evidence in support of our icing observations can be found in the Supporting Information (cf. S.1. and Figure S1).

By contrast, in the absence of metal ions, the water molecules took a substantially longer time to organize under identical conditions. Figure 3d illustrates a snapshot taken at 150 ns in the absence of the metal ions. It is apparent that all water molecules are not perfectly aligned in the hexagons. We further extended our simulation to 500 ns but observed no substantial transformation from water to polar cubic ice over the initial structure,^4a,4f,20^ even after 150 ns. This is also the conclusion one reaches from the corresponding RMSD plot in Figure S2c, at which we observe a constant pattern of deviation almost after 130–150 ns of simulation. In contrast, a similar constant pattern of deviation (cf. Figure S2a,b) is seen for metal-containing systems almost from the start of the production MD simulation, which again suggests a quick process (∼10 ns for Al^3+^ or ∼14 ns for Mg^2+^) of the formation of ice hexagons. Indeed, a mere visual comparison also clearly demonstrates that water molecules are more aligned in Figure 3b,c than in Figure 3d, and the alignment that occurs in the presence of metal ions requires a much shorter simulation time (5–10 ns in Figure 3b and 9–14 ns in Figure 3c vs 150 ns in Figure 3d). In conclusion, therefore, the metal ion acts as a long-range organizer of the water molecules and aids thereby in the speedy development of the ice-like hexagons.

**Figure 4:**
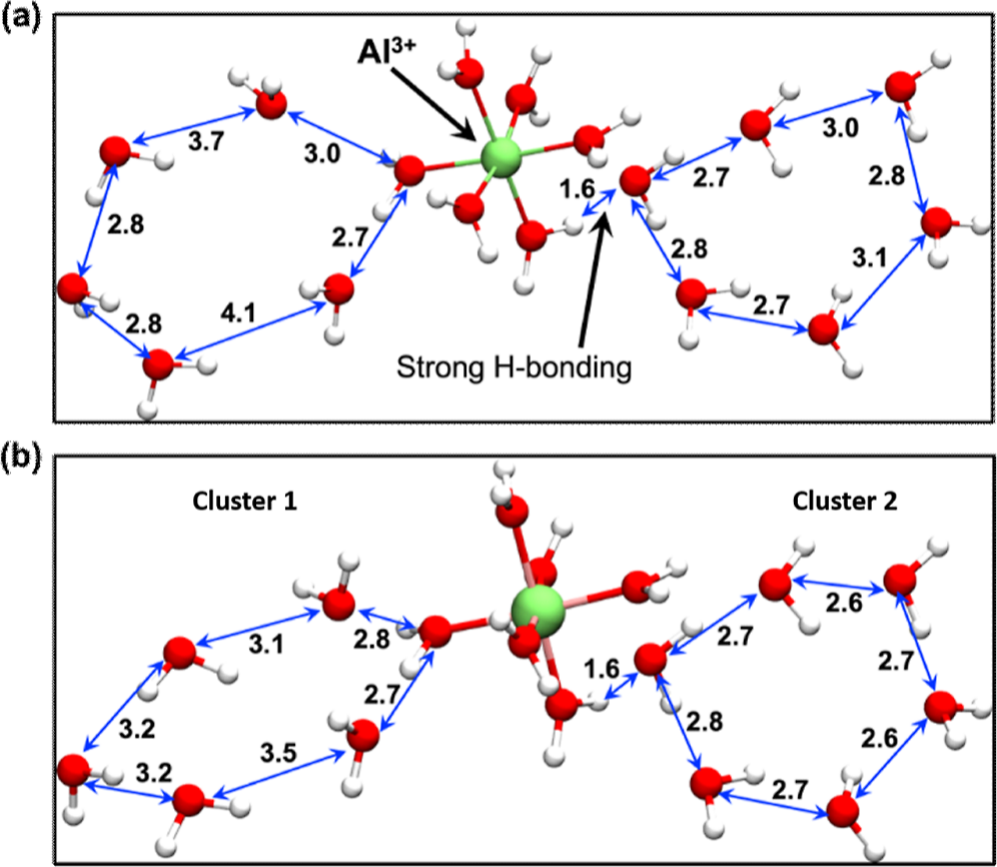
Two types of ice-hexagon geometries differently connected with the Al^3+^ ion. (a) Before forcefield minimization and (b) after forcefield minimization. The double-headed blue arrow denotes the end-to-end oxygen atom distances with the nearest water molecule. All distances are in Å units.

A closer look at the MD trajectory in the presence of Al^3+^ ions further (Figure 4a) reveals that the formation of ice-like hexagon clusters can be of two types, depending on their origin from the surface of the Al^3+^ ion. In order to obtain the lowest energy structures, we performed energy minimizations of these two-crystal geometries using an identical forcefield and obtained the geometries shown in Figure 4b. Thus, the two distinct types of ice-like hexagon clusters are clusters **1** and **2** in Figure 4b. One type (**1**) involves a hexagon, which grows via two hydrogen bonds to a single H_2_O molecule that is ligated to Al^3+^. The second type (**2**) evolves by a single hydrogen bond to a water ligand of the Al^3+^ ion. As such, in both cases, Al^3+^ is hexacoordinated as [Al(H_2_O)_6_]^3+^ by a primary coordination shell, which gives rise to a two-way growth of water hexagons.

**Figure 5:**
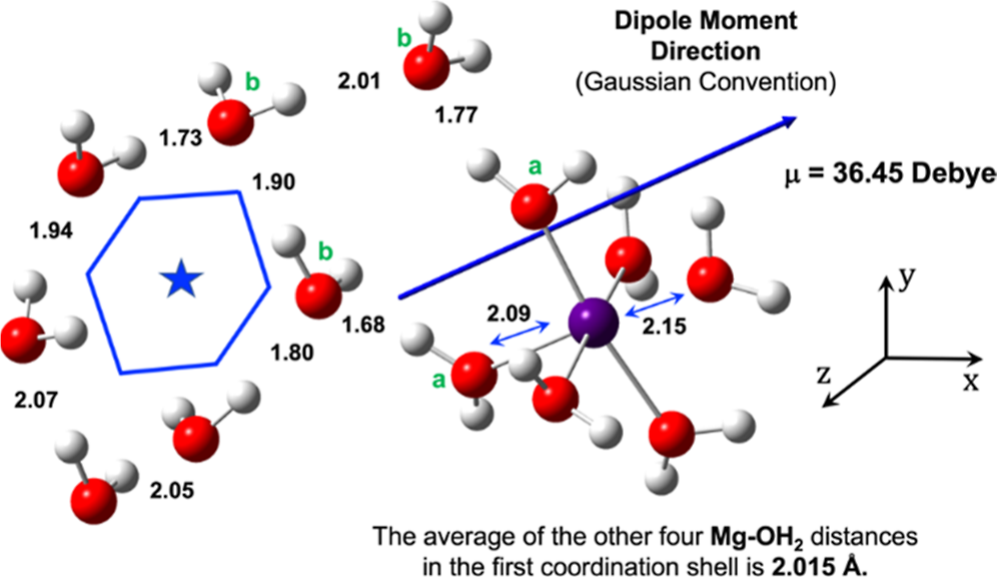
Ice-hexagon geometry connected with the [Mg(H_2_O)_6_]^2+^ unit. The double-headed blue arrows denote the distances between the Mg^2+^ ion and the coordinated H_2_O molecules. The distances in the absence of the double-headed blue arrow denote the hydrogen bonding distances between the oxygen and the hydrogen of the nearest water molecules. All distances are in Å units. The quantum mechanically calculated dipole moment vector (using DFT B3LYP/Def2-TZVP) for the [Mg(H_2_O)_6_]^2+^ and H_2_O hexagons.

Similarly, we further investigated the role of the Mg^2+^ ion in propagating the ice hexagon from its surface at a molecular level. Although we have seen that water molecules are hexagonally attached to Mg^2+^, the Mg^2+^-water distances are not identical, with two axial water molecules having slightly longer distances (2.09 and 2.15 Å) than the other four, ca. 2.015 Å (see the double-headed blue arrow in Figure 5). Furthermore, the [Mg(H_2_O)_6_]^2+^ system is more loosely packed compared with [Al(H_2_O)_6_]^3+^, as evidenced by the radial distribution function (RDF) plots of both systems in Figure S3. The RDF plots for both systems also quantitatively depict the distribution of water molecules surrounding the Mg^2+^/Al^3+^ ions and show that the coordination number for both systems is 6.

Furthermore, the origin of the ice hexagon on the surface of [Mg(H_2_O)_6_]^2+^ is different from that of [Al(H_2_O)_6_]^3+^; for [Mg(H_2_O)_6_]^2+^, we do not see the two distinct types of crystal geometries which were observed for [Al(H_2_O)_6_]^3+^. Thus, as shown in Figure 5, we observe that in the direction of the applied electric field, three water molecules (marked as “b”) interact with the two coordinated water molecules (marked as “a”) and form a pseudo-hexagon involving the Mg^2+^ ion. This pseudo-hexagon further organizes other water molecules and assists in the propagation of ice-like-hexagons in all three directions but more prominently along the electric field axis (cf. Figure 3).

### Nucleation of 3D-Ice Structures

Let us try to comprehend how the metal ions (Al^3+^ and Mg^2+^) could contribute to the generation of ice-like hexagons. Thus, since water molecules have significant dipole moments, they get attracted by the charged metal ions, which provide hexagonal mini surfaces for [Al(H_2_O)_6_]^3+^ and [Mg(H_2_O)_6_]^2+^ that act as “seeding” mediators upon which the entire ice crystal builds up. In contrast, the ion-free system does not have such a support from which water can grow as a crystalline phase. Again, from the perspective of classical nucleation theory, if a foreign particle can stabilize the crystallized ice phase, then it also catalyzes the process of the (water → ice) transformation.^5b^

As such, it is evident from Figures 4 and 5 as well as the preceding discussion how the metal ions can serve in the ice-making process and assist in the growth of 3D ice crystals. As we saw above (Figures 4 and 5), once the [Al(H_2_O)_6_]^3+^ and [Mg(H_2_O)_6_]^2+^ are formed, they start to structure the surrounding water molecules via the LEFs of the metal center and of the O–H bonds of its ligated water molecules and of the water molecules in the initially formed hexagons (Figures 4b and 5), which emanate by interacting with [Al(H_2_O)_6_]^3+^ and [Mg(H_2_O)_6_]^2+^. Each water molecule in such a hexagon has pseudo-equatorial and pseudo-axial O–H bonds, which act as LFEs that can propagate the water hexagon formation in 3D.

As seen in Table 2, this nuclear ice structure has LEFs in all directions. Hence, it will give rise to 3D-ice formation. Since the LEF_*X*_ is the largest, the ice will grow fastest in the *X* direction and more slowly in the *Y*,*Z* directions.

**Figure 6:**
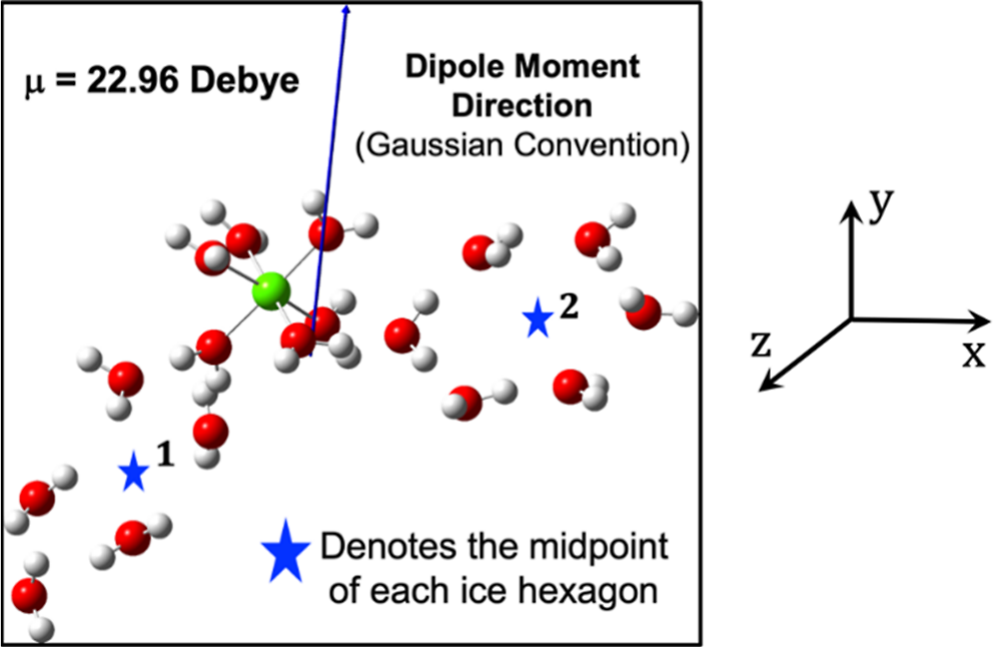
Quantum mechanically calculated dipole moment vector (using DFT B3LYP/Def2-TZVP) for the [Al(H_2_O)_6_]^3+^ and H_2_O hexagons of Figure 4b. Note that the geometry in Figure 3b is slightly tilted to properly depict the perpendicular direction of the dipole moment vector.

To support these conclusions, we calculated quantum mechanically the total LEF of the metal ions and the water hexagons (Figure 5 and 6), in the absence of any external electric field. These LEFs are shown in Table 2.^21^

In this section, we have described how the LEF, which originates from the positively charged metal ion, contributes to organizing the water molecules into ice-like structures in all three directions. Similarly, it is also important to understand that the same metal ion can modify the interaction between water molecules and the external electric field in its vicinity.

## Discussion

A search for new “ice-maker” ions from among the hydrated metallic ions found that hydrated octahedral Al^3+^ and Mg^2+^ ions induce electro-freezing at −4 °C with 100 and 40% probabilities, respectively. These findings are supported by MD simulations, which show that these ions can form “ice-like” hexagons and induce ice nucleation, and Al^3+^ does so more efficiently than Mg^2+^. In contrast, experiments show that the octahedral ions of Na^+^ do not affect the electro-freezing process. These results can be rationalized by two effects that operate in tandem. First, the stereochemistry of the coordinated water molecules around the different metallic ions and, second, the degree of polarization that each metal ion impinges on those water molecules. Since all these three hydrated ions assume a similar octahedral configuration, it suggests that the different electro-freezing properties induced by the three metallic ions result from the differences in polarization they apply to the coordinated water molecules. The degree of polarization depends on the valency of the ion as well as the volume of its nucleus. The different degree of polarization of the coordinated water molecules results in differences in their pH. This value can be inferred from the measured pH of solutions of the metal ions pH ∼ 2–3 for Al^3+^, pH ∼ 5–6 for Mg^2+^, and pH ∼ 7 for Na^+^. Accordingly, the interactions of the coordinated waters with the surrounding conjugated basic water should differ for the three ions. In order to induce electro-freezing, these interactions must lead to the creation and stabilization of “ice-like” hexagons. In the case of the most acidic coordinated water molecules of Al^3+^, the binding properties should be superior in comparison to the Mg^2+^. The coordinated water molecules around Na^+^ assume similar properties to those of bulk water. Waluyo et al. showed by X-ray–Raman investigations a similar correlation between the binding strength of those three ions with water molecules in the hydrated water sphere.^10^

In this system, the hydrated positive metal cation is attracted to the negatively charged cathode. At the cathode, the interfacial water layer becomes ordered with the hydrogen atoms pointing toward the surface, and there is the formation of hydroxide ions due to the electrolysis of water.^19e^ The role of the electric field is to attract the hydrated cation and concentrate around its ordered water molecules and hydroxide anions, where it can form not only an ordered structure but the “ice-like” hexagons necessary to induce ice nucleation. This process leads to an elevation in the freezing temperature of SCW. In addition, close to the surface, the electric field is the greatest, and it can distort the polarization of the hydrated cation.

## Conclusions

In accordance with our previous work^5a^ as well as the work of others,^22^ the present experiments with Al^3+^ and Mg^2+^ strengthen our hypothesis that the electro-freezing of SCW involves a chemical process. We found that different chemical species can initiate ice nucleation on pyroelectric surfaces as well as electrolytic cells, as shown here. These species are called kosmotropic “ice makers”. In particular, Al^3+^ and Mg^2+^ cations have the ability to behave as “ice-making” ions. The comparison between Al^3+^, Mg^2+^, and Na^+^ ions allows us to separate the polarization and geometric effects. We showed that the ability of the cation to polarize the surrounding water molecules dictates the ion’s “ice-making” ability. In order to generalize this mechanism, fourth-row transition metals with similar polarization and geometry should be investigated.

The “ice-making” ions arrange not only the closest water molecules but also affect the long-range order of the water, meaning that they are kosmotropic. This work shows that ice nucleation is another property which can be induced by kosmotropic ions, similar to the Hofmeister effect in biology. It influences the ability of water molecules to arrange in “ice-like” hexagons.

In addition, we anticipate that the ability of Al^3+^ and Mg^2+^ to induce ice nucleation might explain the icing behavior of metallic electrodes made of Al and Mg.^19b,19c^ Even more so, this work can contribute to our understanding of how minerals containing Al^3+^ and Mg^2+^ can induce ice nucleation.

## Materials and Methods

### Sample Preparation

Ni electrodes were fabricated using photolithography (photoresist S1805, MA/BA6 Karl-Suss mask aligner) followed by e-beam deposition (Telemark) of 10 nm Ti (99.999% purity) and 200 nm Ni (99.999% purity) on a *c*-plane sapphire wafer and lift-off.

An insulating layer made from the photoresist AZ4562 was obtained by photolithography.

Metal solutions were made by first making a 10^–1^ M solution with the appropriate nitrate salt (aluminum nitrate nonahydrate 99.997% purity Sigma-Aldrich, magnesium nitrate hexahydrate 99.99% purity Suprapur, sodium nitrate 99.99% purity Suprapur), and serial dilutions were performed to achieve 10^–5^ M solutions.

### Monitoring of the Electro-freezing Experiments

To determine the SCW freezing point, a 1 μL droplet of ultrapure distilled water (18.2 Ω cm) was placed on the sample. The sample was then cooled down on a Peltier stage from room temperature down to the target temperature of the measurement using an INSTEC mK2000 temperature controller. At this temperature, a pulse of −50 V was applied, and freezing/no freezing was observed optically through a light microscope (Zeiss AXIO Imager.M2.m). After the freeing event, the sample was heated slowly (0.5 °C/min) back to room temperature in order to monitor the melting point. A K-type thermocouple connected to a Keithley 2110 5 1/2 Digit Multimeter was used to measure and record the temperature of the sample during the experiments. The correction of the melting point to 0 °C and the freezing point accordingly is used to eliminate artificial shifts in measured temperature that originate from the thermocouple. The freezing temperature was monitored by a light microscope (Zeiss AXIO Imager.M2.m) connected to a complementary metal–oxide semiconductor BlueFOX3 camera.

## Computational Methodology

### System Preparation and MD Simulation

An aluminum ion (Al^3+^) was packed inside randomly distributed water molecules using the PACKMOL program^23^ (see Figure 3a) in Amber 18.^24^ To optimize the density of water required for ice nucleation, we first prepared three different systems, each with a different number of water molecules: 690, 700, and 710. Note that all three different numbers of water molecules were packed in a fixed dimension (26.3 × 26.1 × 28.5 Å^3^) of the simulation box. Once we obtained the optimized system with the Al^3+^ ion, it was then substituted with the Mg^2+^ ion and subjected to geometry optimization in order to perform similar calculations with the Mg^2+^-containing system. While modeling the Al^3+^ and Mg^2+^ ions, we took the parameters from the work of Li et al.,^25^ which overcome the drawbacks^26^ of the modeling of highly charged ions (Al^3+^) and water using the pair potential approximation. A brief discussion is also added in Supporting Information (cf. S.4.).

In this study, we prefer the TIP4P/2005 forcefield^27^ parameters over the TIP4P/Ice forcefield^28^ for the simulation of water molecules. A brief description of this preference is included in Supporting Information (cf. S.5.). However, to remove the ambiguity, we repeated the simulations employing the TIP4P/Ice forcefield, keeping all other variables identical. Nevertheless, the TIP4P/Ice forcefield practically produces similar results at 270 K as TIP4P/2005 does at 250 K.

After completing the system setup, the water-ion mixture system was minimized using 5000 steps of steepest descent followed by 5000 steps of conjugate gradient algorithm. The system was then gently heated to 273 K for 50 ps using the *NVT* ensemble, followed by the use of 1 ns at the *NPT* ensemble at a target temperature of 273 K and a pressure of 1.0 atm using the Langevin thermostat^29^ and Berendsen barostat^30^ with a collision frequency of 2 ps and a pressure relaxation time of 1 ps. The so-generated equilibrated systems underwent a further production run in the *NVT* ensemble for a maximum of 500 ns in the presence and absence of the external electric field. These runs used a multitrajectory approach in which the simulation was restarted at a random velocity after the completion of each 50 ns duration. In all cases, we performed three replica simulations to remove the probable false implications. Note that the target temperature mentioned in the simulation protocol is case-sensitive, as we performed different sets of calculations with varying temperatures.

The Monte Carlo barostat was used during all production MD simulations.^31^ The SHAKE algorithm^32^ was employed to constrain the hydrogen bonds, while particle mesh Ewald^33^ and appropriate cut-off distances (6 Å) were used to treat the long-range electrostatic and van der Waals forces.

The GPU version of the AMBER18 package^24^ was used to carry out all MD simulations. The AMBER18 in the built CPPTRAJ module^24^ was employed to analyze all the results.

## Supporting Information Available

Results of the icing experiments with 10^–5^ M Mg(NO_3_)_2_ at different temperatures compared with pure water, RMSD plots corresponding to a few relevant MD simulations, orientational tetrahedral order parameter plots, and justifications and arguments in support of our employed methods and obtained results (PDF)

## Abbreviations

SCW: supercooled water
MD: molecular dynamics
