# Supplementary material for: Electro-Freezing of Supercooled Water Is Induced by Hydrated Al3+ and Mg2+ Ions: Experimental and Theoretical Studies
Source: J Am Chem Soc. 2023 Aug 21;145(34):18904–11. doi: 10.1021/jacs.3c05004 (PMC10472506; doi:10.1021/jacs.3c05004)
Supplement: Supplementary file 1 — ja3c05004_si_001.pdf [file ja3c05004_si_001.pdf]

Supporting Information

Electro-freezing of Supercooled Water Is Induced by Hydrated Al^{3+} and Mg^{2+} ions: Experimental and Theoretical Studies

Leah Fuhrman Javitt[‡¤], Surajit Kalita[‡¤¤], Kshatresh Dutta Dubey[¤¤¤], David Ehre[¤], Sason Shaik[¤¤*], Meir Lahav[¤*] and Igor Lubomirsky[¤*]

[¤]Department of Molecular Chemistry and Materials Science, Weizmann Institute of Science, Rehovot 7610001, Israel [¤¤]Institute of Chemistry, The Hebrew University of Jerusalem, Edmond J. Safra Campus, Givat Ram, Jerusalem 9190401, Israel

[¤¤¤]Department of Chemistry, School of Natural Sciences, Shiv Nadar University, Greater Noida, Uttar Pradesh 201314, India

Table of Contents:

Table S1: Experimental data from the icing experiments with ions and pure H_2O S2

S.1. Statistical Evidence of Ice Nucleation Derived from MD Simulations Trajectory S3

S.2. Interaction of Water and External Electric Field in the Vicinity of Metal Ion S4

S.3. Modeling of the Interaction of Highly Charged Metal Ions and Water Molecules S5

S.4. Preference of TIP4P/2005 Forcefield over TIP4P/Ice Forcefield S6

Figure S1. Orientational Tetrahedral Order (OTO) parameter plot S3

Figure S2. RMSD plot S6

Figure S3. Radial Distribution Function (RDF) plot S7

References. S7

Table S1: Results from the icing experiments with Al^{3+}, Mg^{2+}, Na^{+}, and pure water at different temperatures

10^{-5} M $Al(NO_3)_3$	T	# froze	# measured
	-4.5	6	6
	-4	20	20
	-5	2	2
	-6	2	2
		30	30
For Al histogram	-3	0	11

10^{-5} M $Mg(NO_3)_2$	T	# froze	# measured
	-4	5	14
	-4.5	6	13
	-5	7	18
		18	45
For Mg histogram			
	-2	0	11
	-3	0	11
	-6	4	10
	-7	1	2
	-8.5	4	9

10^{-5} M $Na(NO_3)$	T	# froze	# measured
	-6	0	25

pure H_2O	T	#froze	# measured
	-6.3	0	22
	-7.3	1	24
	-8.3	1	27
	-9.5	2	3
	-11	3	5
	-12	7	7

S.1. Statistical Evidence of Ice Nucleation Derived from MD Simulations Trajectory

One common and mostly used statistical parameter to differentiate the ice like structure from random water molecules is Orientational Tetrahedral Order (OTO) parameter. The OTO value ranges from 0 to 1; in which 0 indicates complete randomness and 1 denotes perfect ordering.

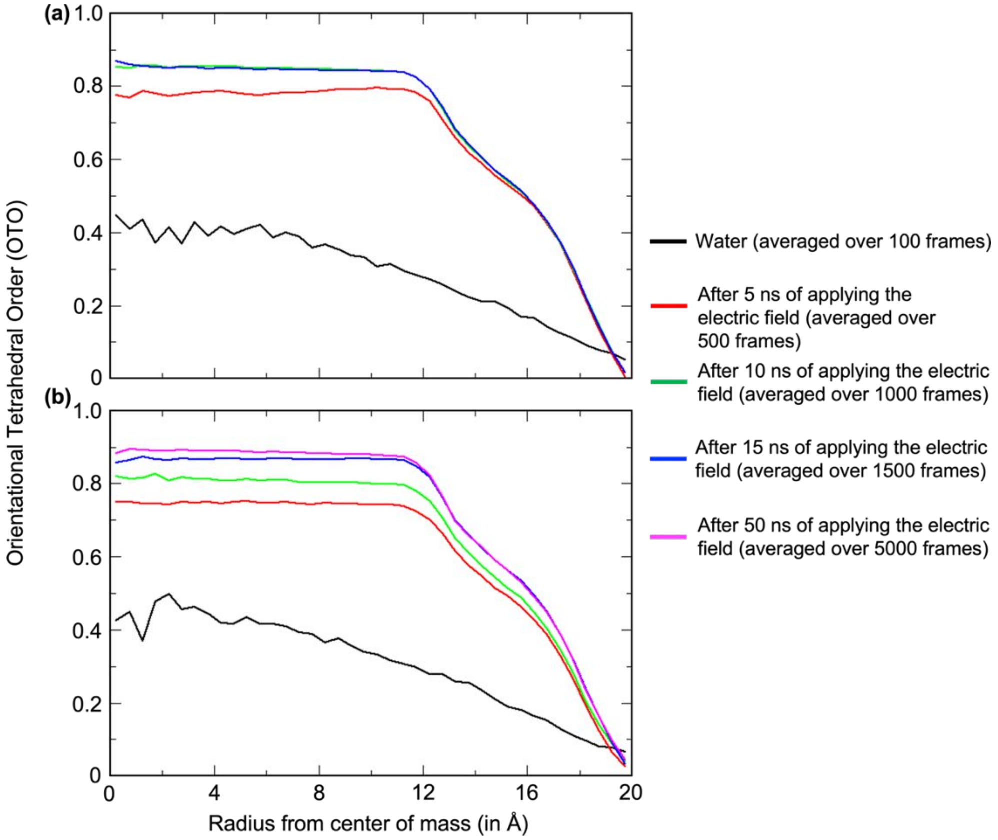

Figure S1: Orientational tetrahedral order parameter for (a) Al^{3+} -- water system and (b) Mg^{2+} -- water system.

Let us then analyze figure S1. We can see that before employing the electric field, the water distribution is fully random (black curve), with an OTO value of roughly ~0.4. However, as soon as an electric field is applied, water molecules begin to organize themselves. If we compare the OTO values (red curves) after 5 ns, we can see that Al^{3+} water systems are more organized than Mg^{2+} water systems. In the case of the Al^{3+}, the system reaches the maximum ordered arrangement after 10 ns (green curve) and exhibits the same pattern after 15 ns (blue curve). This clearly shows that ice formation is completed after only 10 ns of simulation time, and it remains the same with progress of the simulation.

In the case of the Mg^{2+} -- water system, on the other hand, we observe a considerable increase in OTO value upon moving from 5 ns (red curve) to 10 ns (green curve), as ice-like

hexagons begin to form after 8 ns. After 15 ns of simulation time, the value of OTO rises by another few units, indicating that the buildup of an ice-like hexagon is virtually completed. Similarly, the OTO value climbs slightly until 50 ns (magenta curve) of simulation time in the Mg^{2+} system, which we do not see in the Al^{3+} system (where OTO is nearly identical in 10 and 15 ns of simulation time). This trend provides yet another evidence of the sluggish pace of production of the ice-like hexagons in the Mg^{2+} systems. Consequently, we may conclude that the rate of production of ice-like hexagons is substantially slower in the Mg^{2+} -- water system than in the Al^{3+} one.

S.2. Interaction of Water and External Electric Field in the Vicinity of Metal Ion

Let us look at the Figure 3d wherein the ice hexagons are induced by the electric field in the absence of metal ions. In this case, all water dipoles are organized themselves in the direction of the applied electric field. On the other hand, when we add the highly positively charged ions, the water molecules are locally polarized by the positively charged metal ions despite the presence of external electric field and form the first hydration shell (cf. Figure 4a and 5). As a result, some of the water dipoles realign themselves against the applied electric field, which modify the interaction of water and external electric field in the vicinity of highly charged positive metal ions. It is now understood that water molecules in the first hydration shell becomes polarized and the water molecules donate some of their lone pair electron density towards the positive metal ions; these water molecules develop a partial positive charge as compared to neutral water molecules (however we cannot estimate the degree of polarization using classical water forcefield). As a result, when comparing to water-water interactions in pure water at pH=7, the interaction between water molecules in the first hydration shell and neighboring water molecules eventually alters. Hence, the intermolecular interaction is modified in the vicinity of charged ions, albeit the presence of a strong external electric field.

S.3. Modeling of the Interaction of Highly Charged Metal Ions and Water Molecules

In the context of ion-water interactions, we primarily focus on the trivalent Al^{3+} ion. TIP4P/2005, like other classical water forcefields, is also a non-polarizable water model, and hence it may be difficult to assess all complicated phenomena generated by the interaction of high valent cation and water molecules. Nevertheless, the Al^{3+} ion parameter has recently been parametrized with

TIP4P-Ew forcefield (these parameters show a perfect transferability with the TIP4P/2005 water model according to the recent report by Döpke et. al[1] and it reflects a reliable correlation with various experimental data, such as hydration free energy (HFE), experimental ion-oxygen distance (IOD), coordination number (CN), etc. In fact, these forcefield parameters can generate the experimental HFE and IOD values within the error margin of ±1 kcal/mol and ±0.01 Å, respectively. The parameters employ a 12-6-4 LJ type non-bonded model during the ion parametrization and address the problem that occurs with a 12-6 LJ type non-bonded model. In general, the 12-6 LJ type non-bonded model highly underestimate the experimental properties with increasing metal charges. Thus, while a complete picture of ion-water interactions can be achieved with *ab-initio* quantum simulations, nevertheless we believe that our interaction model is sufficiently good to qualitatively describe the Al^{3+} -- water interactions with moderate computational cost.

S.4. Preference of TIP4P/2005 Forcefield over TIP4P/Ice Forcefield

Despite the use of TIP4P/Ice forcefield is growing for ice simulations, there are several other works which employed the TIP4P/2005 forcefield for ice-water simulation and obtained the reasonable results[2]. In fact, a study by Noya et. al.[3] claims that the TIP4P/2005 water model exhibits slightly better results than the TIP4P/Ice forcefield, for some of the experimental properties of ices (Ih, II, III, V and VI), such as equation of state, thermal expansion coefficient and isothermal compressibility. Similarly, Aragones et. al.[4] determines the complete phase diagram of TIP4P/2005 water model **in the presence of an external electric field** and discusses all aspects of ice formation and disappearance with varying conditions. Furthermore, the TIP4P/Ice forcefield was developed in an intent to obtain the properties of ice and amorphous water. As such, the water molecule under this forcefield would have a natural tendency to form ice-structures. Nevertheless, we are trying to understand the role of positively charged ions in ice nucleation from water, and

our results match the experimental findings. Thus, we believe that TIP4P/2005 water forcefield is suitable to the objective of our research problem.

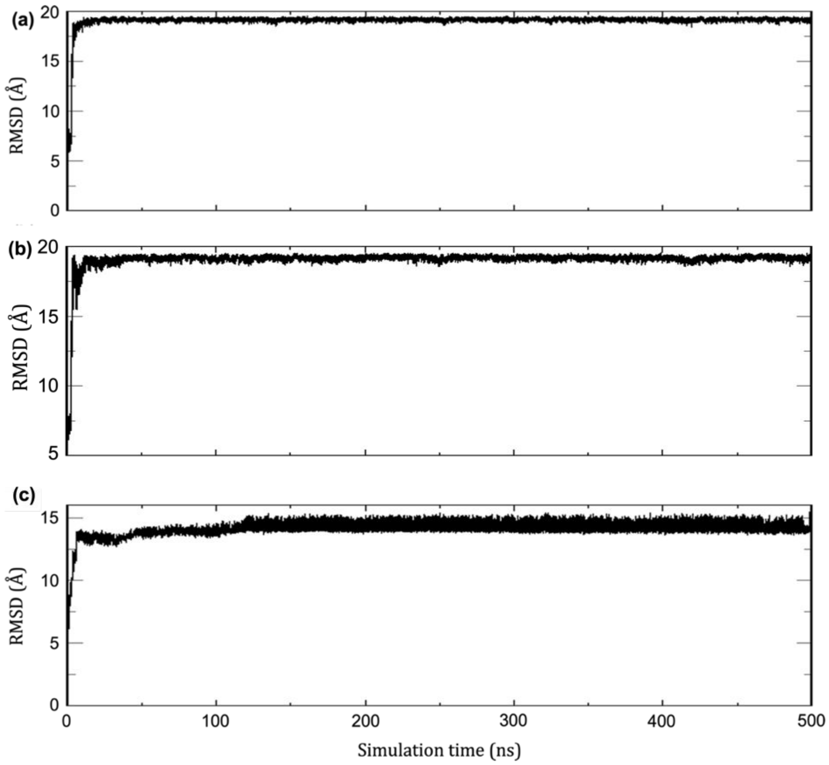

Figure S2: RMSD plots correspond in the MD trajectories (a) in the presence of Al^{3+} ion and, (b) in the presence of Mg^{2+} ion, (c) in the absence of metal (Al^{3+} and Mg^{2+}) ions.

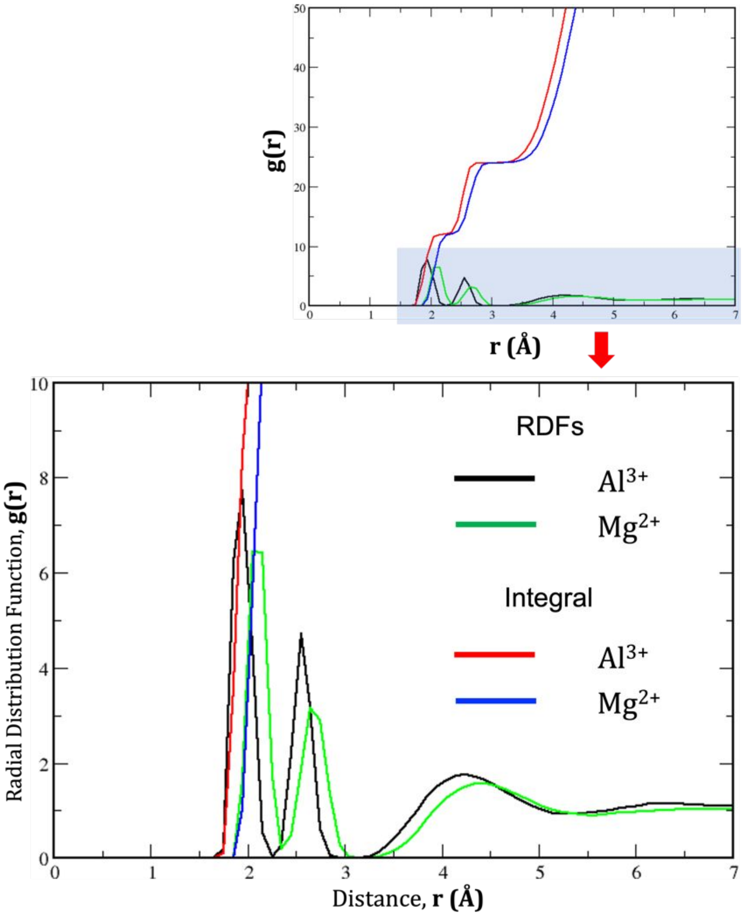

Figure S3: The radial distribution function and the integral plot considering the metal ions as reference point.

References:

1. Döpke, M. F.; Moultos, O. A.; Hartkamp, R., On the transferability of ion parameters to the TIP4P/2005 water model using molecular dynamics simulations. *The Journal of chemical physics* **2020,** *152* (2).

2. (a) Benet, J.; MacDowell, L. G.; Sanz, E., A study of the ice–water interface using the TIP4P/2005 water model. *Physical Chemistry Chemical Physics* **2014,** *16* (40), 22159-22166; (b) Espinosa, J. R.; Vega, C.; Sanz, E., Ice–water interfacial free energy for the TIP4P, TIP4P/2005, TIP4P/ice, and mW models as obtained from the mold integration technique. *The Journal of*

Physical Chemistry C **2016,** *120* (15), 8068-8075; (c) Aragones, J.; Conde, M.; Noya, E.; Vega, C., The phase diagram of water at high pressures as obtained by computer simulations of the TIP4P/2005 model: The appearance of a plastic crystal phase. *Physical Chemistry Chemical Physics* **2009,** *11* (3), 543-555.

3. Noya, E.; Menduina, C.; Aragones, J.; Vega, C., Equation of state, thermal expansion coefficient, and isothermal compressibility for ices Ih, II, III, V, and VI, as obtained from computer simulation. *The Journal of Physical Chemistry C* **2007,** *111* (43), 15877-15888.

4. Aragones, J.; MacDowell, L.; Siepmann, J.; Vega, C., Phase diagram of water under an applied electric field. *Physical review letters* **2011,** *107* (15), 155702.
